# Study on Thermal-Oxidative Aging Properties of Ethylene-Propylene-Diene Monomer Composites Filled with Silica and Carbon Nanotubes

**DOI:** 10.3390/polym14061205

**Published:** 2022-03-17

**Authors:** Xiaoming Zhang, Jian Li, Zilong Chen, Ce Pang, Shaojian He, Jun Lin

**Affiliations:** State Key Laboratory of Alternate Electrical Power System with Renewable Energy Sources, North China Electric Power University, Beijing 102206, China; zxm66770912@163.com (X.Z.); li1270098939@163.com (J.L.); zilongchen86@163.com (Z.C.); pc_1232021@126.com (C.P.)

**Keywords:** ethylene-propylene-diene monomer, carbon nanotubes, mechanical properties, thermal-oxidative aging

## Abstract

In this work, a small amount of carbon nanotubes (CNTs) was used to partially replace the silica in ethylene-propylene-diene monomer (EPDM) to prepare EPDM composites via mechanical blending. The mechanical properties, thermal-oxidative aging properties and thermal stability of the composites were systematically investigated. The results showed that with the increase of CNTs content, the Shore A hardness and stress at 100% strain of the composites increased, while the elongation at break decreased. With the aging time increasing, the aging coefficient and elongation at break of composites decreased while hardness increased due to the raise of crosslinking density. In addition, evidences were found to demonstrate the improved aging resistance by adding CNTs in the EPDM composites, including the less change in Shore A hardness, the smaller change ratio of elongation at break and the lower aging coefficient. When the content of CNTs reached 10 phr, the aging coefficient of the EPDM composite aged for 168 h was nearly twice that of the composite without CNTs, and the thermal stability of the EPDM composite with CNTs was improved as demonstrated by thermal analysis.

## 1. Introduction

Ethylene-propylene-diene monomer (EPDM), one of the most commonly used rubbers, has been widely used in low/medium voltage, high voltage and even ultra-high voltage cables due to the excellent electrical insulation, good ozone resistance, moisture resistance, heat resistance, cold resistance and other properties [1,2,3,4,5]. However, EPDM is still prone to aging under the combined action of heat, oxygen, light and other conditions, resulting in a significant decline in performance [6,7,8]. So far, anti-aging agents were commonly applied to improve the aging resistance of rubbers [9,10,11,12,13]. With respect to rubber, the essence of anti-aging is to prevent the degradation and crosslinking of rubber by using suitable additives to trap free radicals formed by rubber components and thus to delay the attack of free radicals on rubber macromolecular chains.

The commonly used anti-aging agents are antioxidants, including N-isopropyl-N’-phenyl-p-phenylene diamine (4010NA), 2-sulfur-benzimidazole (MB), N-4(phenylephenyl)-maleimide (MC) and 2,2,4-thrimethyl-1,2-dihydroquinoline polymer (RD) [8]. However, most of the anti-aging agents like antioxidants are organic compounds, and the production of those compounds might cause environmental problem. Moreover, the above mentioned traditional antioxidants could be lost by volatilization and extraction, or diffusion onto the rubber surface, leading to the decline of the long-term protection effect. Efforts have been made to prevent the loss of long-term protection from antioxidants, like sustained release of antioxidant from inorganic fillers [14,15], but usually costly and time-consuming.

In contrast, the inorganic fillers that possess the anti-aging function are more efficient to be applied in rubber industry, which also play a role as reinforcing filler [16,17,18,19,20]. Therefore, there has been much academic and industrial interest in the development of rubber nanocomposites filled with fillers that could improve the aging resistance, such as clay, carbon nanotubes (CNTs) and graphene, as a substitute for the traditional fillers including carbon black or silica [21,22,23,24,25,26,27,28,29]. CNTs is the most attractive filler and most intensively investigated among them due to its excellent radical scavenging ability. Ata et al. [30] reported that the heat-resistivity of fluorinated rubber was improved from 200 °C to 340 °C by adding 1 wt% of CNTs. Guan et al. [31] reported the less change in tensile strength and elongation at break by adding CNTs in fluorosilicone rubber. Xu et al. [32] reported that 0.1~0.5 wt% CNTs could block the auto-oxidation degradation of fluorosilicone rubber by scavenging the alkyl radicals. Shimizu et al. [33] reported the rubbery elasticity of the silicone rubber with CNTs was well retained after 7 days at 280 °C, while at same condition the pristine silicone rubber became brittle only after one day.

EPDM based on ethylidene-2-norbornene (ENB) is very sensitive to thermal oxidation, it is expected the addition of CNTs in EPDM would improve the thermal-oxidative aging performance of EPDM composites, which was rarely reported to best of our knowledge. Therefore, in this work, a small amount of CNTs was used to partially replace the silica in EPDM to prepare EPDM composites via traditional mechanical blending method. The variations of mechanical properties for EPDM composites were evaluated after aging for different times at evaluated temperature. The tensile fractured surface morphologies, crosslinking density and thermal stability of EPDM composites were systematically studied. In addition, the thermo-oxidative aging mechanism of EPDM, especially the positive effect of CNTs, was discussed.

## 2. Materials and Methods

### 2.1. Materials

EPDM 4045 (vinyl content of 49~55 wt%, ENB content of 6.8~8.7 wt%) was provided by Jilin Chemical Industry Co., Ltd., Jilin, China. Fumed silica (HDKRV15) was purchased from Wacker Chemie AG, Munich, Germany. CNTs (Flotube 9000, purity: >98%, average length: ~10 μm, tap density: 0.03~0.15 g·cm^−3^) was bought from Beijing Cnano Technology Ltd., Beijing, China. Other ingredients were purchased from the chemical reagent shop of Beijing, China.

### 2.2. Sample Preparation

Silica, CNTs and other additives were subsequently mixed into the gum EPDM for 25~30 min by a standard procedure in a 6-in. two-roll mill (XK-160, Huahan Rubber & Plastic Machinery Co. Ltd., Dalian, China) at room temperature to prepare EPDM/silica/CNTs compound. The compound was then cured in a standard mold using hydraulic hot press (XLB-D 350, Huzhou Dongfang Machinery, Co., Ltd., Huzhou, China) at 160 °C and 15 MPa for optimal curing time (determined by MDR-2000, Dejie Machinery Co. Ltd., Shanghai, China) to obtain EPDM/silica/CNTs composites. The detailed recipe of the prepared EPDM/silica/CNTs composites is presented in Table 1.

### 2.3. Measurements

The morphologies of the CNTs powder and the tensile fracture surface of composites were observed on a Hitachi S-4700 scanning electron microscope (SEM) (Hitachi, Tokyo, Japan) with an acceleration voltage of 20 kV. The power samples were glued onto the observation stand with double-sided tape. All the samples were sputtered with gold before observation.

Mechanical property measurements were conducted using a GT-TC2000 electrical tensile tester (Gotech Testing Machines Inc., Qingdao, China) at the speed of 500 mm·min^−1^ at room temperature according to ASTM D412 and ASTM D624. Shore A hardness was measured using LX-A rubber hardness apparatus (Liuling Instrument, Shanghai, China) according to ASTM D2240.

Thermogravimetric analysis (TGA) was conducted by STA 449 F5 (NETZSCH, Sable, Germany) under nitrogen atmosphere from room temperature to 600 °C at a heating rate of 20 °C min^−1^.

The thermo-oxidative aging properties of the composites were characterized by the aging coefficient and the change rate of mechanical properties. The dumbbell-shaped specimens were placed in a 401A thermal-oxidative aging oven (Shanghai Laboratory Instrument Works Co., Ltd., Shanghai, China) at 150 °C for various time, and then were kept at room temperature for at least 18 h before further tests. The tensile properties of aged specimens were obtained in the same manner as for the unaged specimens. The aging coefficient was calculated using the following equation:

$$Aging\;coefficient=\left(\sigma_{\mathrm{aged}}\cdot \varepsilon_{\mathrm{aged}}\right)/\left(\sigma_{\mathrm{unaged}}\cdot \varepsilon_{\mathrm{unaged}}\right)$$

where *σ*_aged_ and *σ*_unaged_ are the tensile strength of the aged and unaged specimens, and *ε*_aged_ and *ε*_unaged_ are the elongation at break of the aged and unaged specimens, respectively.

The crosslinking density of was measured by equilibrium swelling method [34] and the equilibrium swelling condition was achieved by suspending cured samples in toluene at 25 °C for 72 h. The swelling ratio *Q* can be calculated by equation:

$$Q=\frac{1}{V_{2}}=1+\frac{\rho_{r}}{\rho_{s}}\left(\frac{m_{2}}{m_{1}}-1\right)$$

where *ρ_r_* and *ρ_s_* are the densities of the rubber and solvent, respectively. *m*_1_ and *m*_2_ are the mass of the sample before and after being swollen, respectively. The crosslinking density *v_s_* (mol·m^−3^) of the sample can be calculated by Flory-Rehner equation:

$$\nu_{s}=-\frac{\ln\left(1-V_{2}\right)+V_{2}+\chi V_{2}}{V_{1}(V_{2}^{1/3}-0.5V_{2})}$$

*χ* is the Flory-Huggins interaction parameter between the rubber and the solvent (0.49 for EPDM-toluene), *V*_1_ is the molar volume of solvent.

## 3. Results and Discussion

### 3.1. Microstructure of CNTs

**Figure 1:**
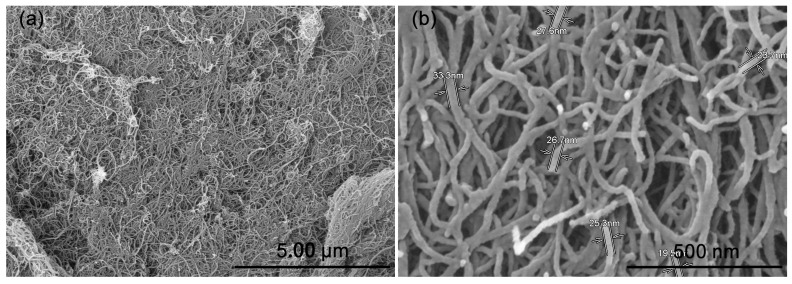
SEM images of CNTs powder: (**a**) low magnification; (**b**) high magnification.

Figure 1 presented the SEM images of the CNTs. It can be seen from the figure that the diameter of CNT is 25 ± 4 nm (by measuring 30 tubes in the image). Due to the intertangling of CNTs, the specific length cannot be accurately distinguished from the SEM image, while it can be confirmed that the aspect ratio of CNTs is very large, which is also observed by previous researchers [35,36,37]. As a result, it is expected that aggregation of CNTs would occur when a large amount of CNTs was incorporated into the rubber matrix [38].

### 3.2. Mechanical Properties of EPDM Composites

Table 2 lists the mechanical properties of EPDM composites filled with various CNTs and silica. With the increase of CNTs content, the Shore A hardness and stress at 100% strain of the composites increase, while the elongation at break decreases. Mechanical properties of rubber composite are generally affected by the difference in the filler-filler interaction, the filler-rubber interaction and the rubber network [18]. Because CNTs is tubular structure with huge specific surface area and high aspect ratio, the filler-filler interaction involving CNTs is very strong. Therefore, the Shore A hardness related to the small deformation mainly affected by filler-filler interaction rises with the increase of CNTs content. During the stretching process, the rubber chains are stretched via slippage and orientation under the influence of filler particles [39], mainly filler-rubber interaction. Compared with the sphere silica, the CNTs with high aspect ratio has a stronger restriction effect on rubber macromolecular chain during the stretching process of composites [21,25], showing a strong filler-rubber interaction. As a result, the stress at a certain strain such as stress at 100% strain becomes higher and the elongation at break goes lower. When the substitution amount of CNTs is small, the tensile strength of the composites is higher than 18.0 MPa, but when the substitution amount of CNT is large (more than 4 phr), the tensile strength decreases, which may be due to the serious aggregation of CNTs and poor dispersion in the rubber matrix.

### 3.3. Microstructure of Tensile Fractured Surface for EPDM Composites

**Figure 2:**
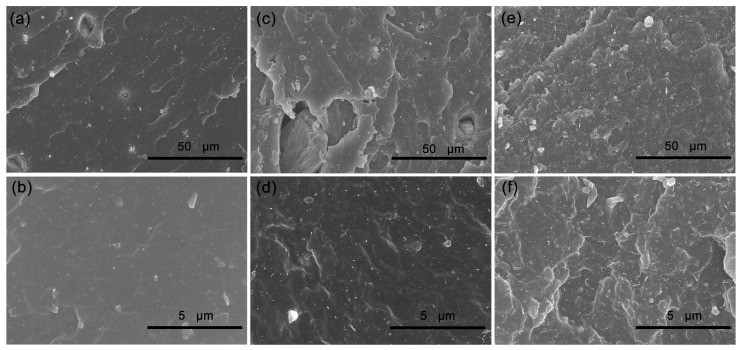
SEM images of tensile fractured surface for EPDM composites: (**a**,**b**) C0; (**c**,**d**) C4; (**e**,**f**) C10.

The SEM images of the tensile fractured surface for C0, C4 and C10 composites are shown in Figure 2. The tensile fractured surface of composites without CNTs is smooth (Figure 2a,b), while the surfaces of composites with CNTs becomes rough (Figure 2c–f), and the more CNTs content, the coarser it becomes. At high magnification, it is clear that the CNTs particles are inserted into EPDM matrix, because the CNTs particles are closely connected with EPDM matrix due to the high aspect ratio, resulting the rougher fractured surface. However, the serious aggregation of CNTs is found for the composite with more CNTs content. Compared with spherical silica nanoparticles, the presence of tubular CNTs makes the composite more prone to generate the stress concentration during the tensile process, so the tensile strength of the composites decrease with the increasing CNTs content.

### 3.4. Aging Performance of EPDM Composites

**Figure 3:**
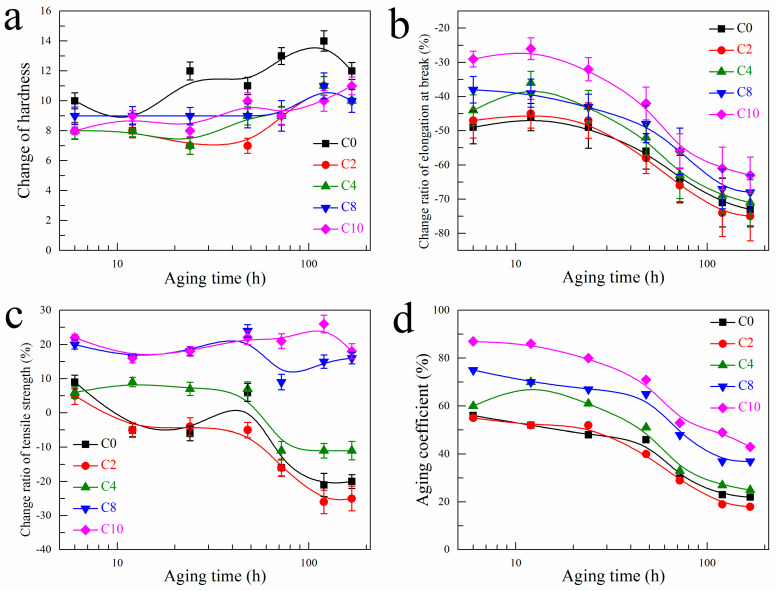
(**a**) Change of Shore A hardness, (**b**) change ratio of elongation at break, (**c**) change ratio of tensile strength and (**d**) aging coefficient for EPDM composites at various aging time.

The aging performance of EPDM composites is evaluated by the change of Shore A hardness, change ratio of elongation at break, change ratio of tensile strength and aging coefficient at various aging time, which are shown in Figure 3a–d, respectively. It is found that the Shore A hardness for all the EPDM composites tend to increase while the elongation at break tent to decrease after aging. The tensile strength is related to the modulus and elongation at break for the composites, so it is possible to observe either decreased or increased tensile strength for the composites. Specifically, the tensile strength for the C8 and C10 composites changes very little during aging, and the tendency of decreased tensile strength for C0 and C2 are found after aging. For C4 composite, the tensile strength first increases and then decreases over the aging time.

**Figure 4:**
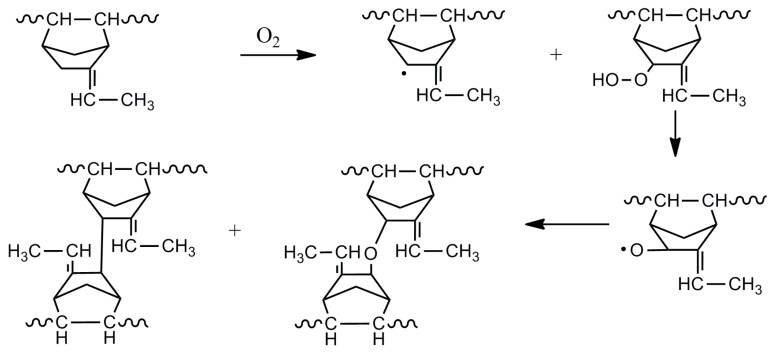
Thermo-oxidative aging mechanism for EPDM.

The increase in hardness and decrease in elongation at break indicate the crosslinking mechanism dominant during the thermal-oxidative aging for the EPDM composites, which is proposed in Figure 4. EPDM containing ethylidene-2-norbornene (ENB) is very sensitive to thermal oxidation. Under high temperature with the existence of oxygen, the chemical bonds with main chains or side groups (RH) can be dissociated to create the free radicals (R) and later to the peroxide radicals (ROO), which further react with labile hydrogens of the rubbers to form unstable hydroperoxides (ROOH). Then the radicals produced from the homolysis of the O–O bond causes the autocatalysis, which steadily abstract or remove hydrogens from the rubber chains to form rubber radicals. At last, antioxidation terminated by the formation of stable structures via the combination of rubber radicals to complete the crosslinking.

In addition, evidences are found to demonstrate the improved aging resistance by adding CNTs in the EPDM composites, including the less change in Shore A hardness, the smaller change ratio of elongation at break and the lower aging coefficient. With the increasing CNTs content, the change ratio of elongation at break mainly decreases while the aging coefficient increases over the aging time. After aging for 6 h, the elongation at break rapidly decreases by 49.0% for C0 composite while only 29.0% for C10 composite. After aging for 168 h, the elongation at break decreases by 73.0% for C0 composite while only 63.0% for C10 composite. Moreover, the aging coefficient of C0 composite decreases to 56.0% and 22.0% after aging for 6 h and 168 h, respectively, while that of C10 composite still remains 87.0% and 43.0%.

**Figure 5:**
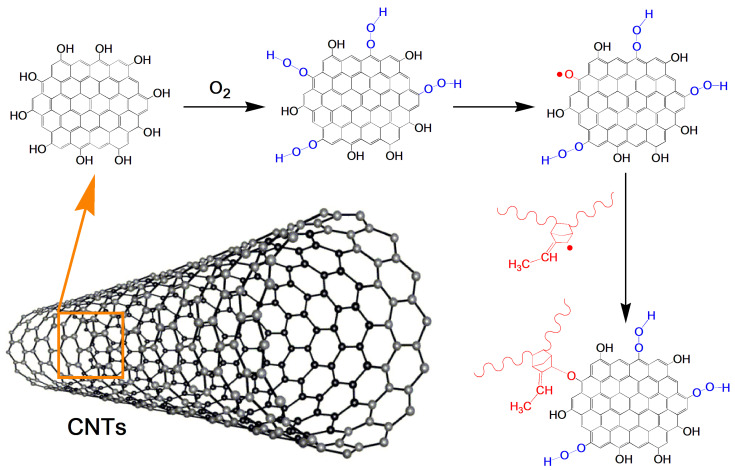
Possible thermo-oxidative aging mechanism for EPDM filled with CNTs.

Since the thermal-oxidative aging of polymeric materials is a radical chain reaction, the antioxidant effects of CNTs is likely to attribute to their radical scavenging abilities. Owing to the strong absorbability of CNTs, groups such as hydroxyl groups on the CNTs surface (containing some hydroxyl groups on the surface of the CNTs Flotube 9000 according to the raw material information) may adsorb active free radicals generated in the aging process of composite materials, and inhibit the post-crosslinking reaction of composite materials, thus effectively improving the ability of EPDM to resist thermal-oxidative aging. The possible reaction mechanism is shown in Figure 5. In the aging process of composite materials with CNTs, hydroxyl groups on CNTs are attacked by oxygen and then oxidized into unstable C-O-O-H, which then decomposes to form C-O∙ that could combine with the active free radicals on rubber macromolecules. Such combination could reduce the content of active free radicals in the rubber composite system to slow down the crosslinking process, so the aging resistance of EPDM would be improved.

### 3.5. Microstructure of Tensile Fractured Surface for Aged EPDM Composites

**Figure 6:**
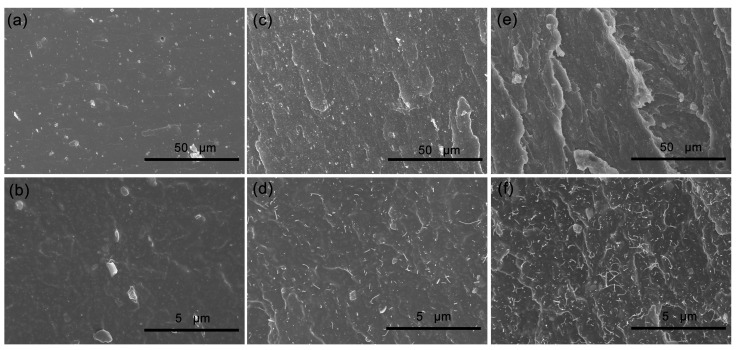
SEM images of tensile fractured surface for aged EPDM composites (aging condition: 150 °C × 72 h): (**a**,**b**) C0; (**c**,**d**) C4; (**e**,**f**) C10.

The SEM images of the tensile fractured surface for the aged EPDM composites are shown in Figure 6. It is found that more and more CNTs are exposed on the surface of the composites, which should be due to the brittleness of the aged composites. In addition, the fractured surface becomes coarser after aging. After aging, due to the free radical capturing effect of CNTs on EPDM macromolecules, more EPDM macromolecules can be bound to CNTs, resulting in the stronger filler-rubber interfacial interaction [40,41], so the tensile fractured surface becomes rougher.

### 3.6. Crosslinking Density of EPDM Composites

**Figure 7:**
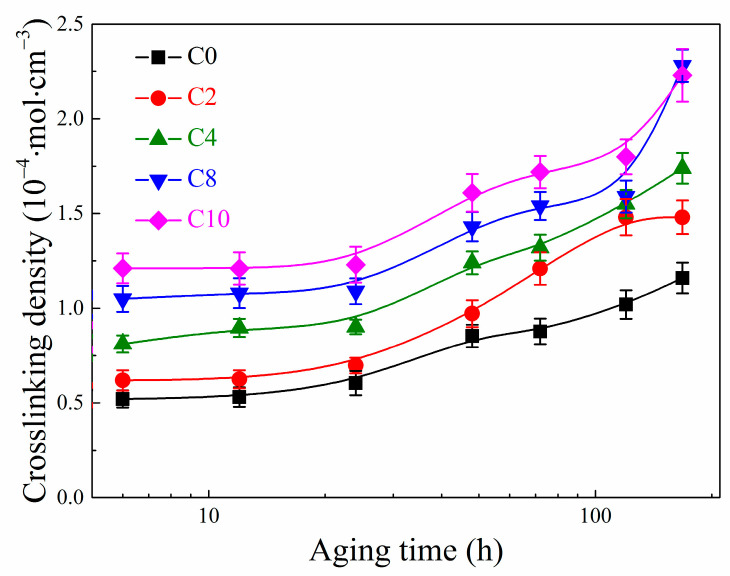
Crosslinking densities of EPDM composites at various aging time.

The degree of thermal-oxidative aging is always characterized by the variation of crosslinking density for vulcanized rubber before and after aging. The crosslinking density of the EPDM composites with various aging time is shown in Figure 7. It can be seen from Figure 7 that, the crosslinking density of composite materials shows an increasing trend with the extension of aging time, which also confirms that the crosslinking reaction of macromolecular chains occurs in EPDM during the thermal-oxidative aging as described above. In addition, it can also be seen from Figure 7 that the crosslinking density of composites containing CNTs is always higher than that of composites without CNTs. Specifically, the crosslinking density rises with the increase of CNTs content at the same aging condition. This is because the specific surface area and aspect ratio of CNT are very large, and CNTs can firmly adhere to the rubber matrix. As compared to the interfacial interaction between silica and EPDM, the interaction between CNTs and EPDM is stronger, resulting in the more extra crosslinking points that increase the total crosslinking density of the composites. This is also an important reason for the higher hardness and lower elongation at break of EPDM composite with more CNTs.

### 3.7. Thermal Stability of EPDM Composites

**Figure 8:**
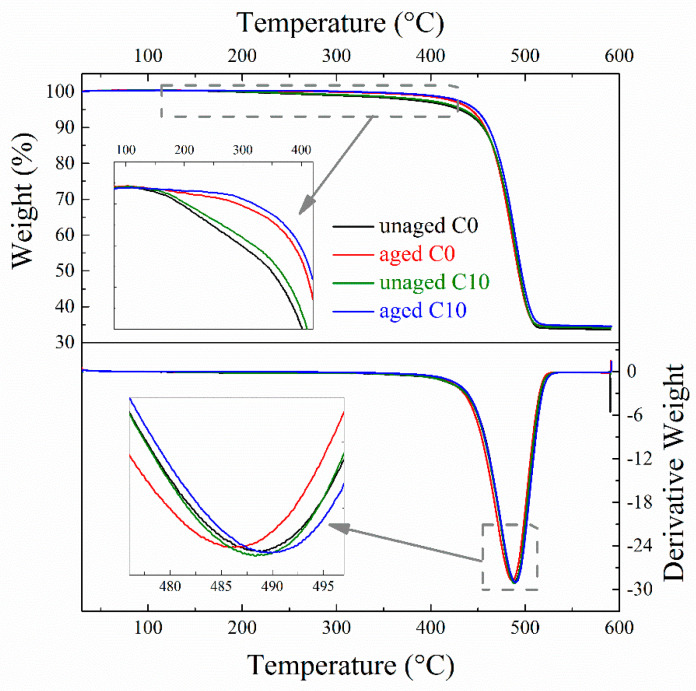
TGA curves of unaged and aged EPDM composites (aging condition: 150 °C × 168 h).

TGA curves of unaged and aged EPDM composites are shown in Figure 8, as well as their derivative weight curves, and the related parameters are shown in Table 3. According to the literature [42], the slight weight loss for EPDM composites at low temperature is due to the volatilization of small molecular ingredients, while the main weight loss should be attributed to the scission of cross-linked EPDM. The temperature at maximum weight loss rate (*T_max_*) for C0 decreases by 2.5 °C after aging, while that of C10 increases by 1.3 °C after aging. Moreover, both the temperature for 5% weight loss (*T*_5%_) and residue mass for C0 are lower than those for C10, indicating the improved thermal stability by adding CNTs. After aging for 168 h, *T*_5%_ for both C0 and C10 composites increases due to the complete volatilization of small molecule compounds during aging, while *T_max_* for the aged C0 composite drops and *T_max_* for the aged C10 composite rises. This indicates that the thermal stability of C10 composite is better than that of C0 composite after aging, that is, improved thermal stability was found for the EPDM composite with CNTs after aging. Such improvement should be also contributed to the combination of CNTs and rubber matrix to achieve stronger interfacial interaction.

## 4. Conclusions

In summary, CNTs was used to enhance the thermal-oxidative aging resistance of EPDM composites. With the increase of CNTs content, the hardness and stress at 100% strain of the composites increased, while the elongation at break decreased. Stronger interfacial interaction was found in the EPDM composites with more CNTs. The increase in hardness and decrease in elongation at break for the EPDM composites indicate the crosslinking mechanism dominant during thermal-oxidative aging, as also proved by the crosslinking density results. Due to the excellent radical scavenging ability, the aging resistance of the EPDM composites was improved by addition of CNTs. After aging for 168 h, the aging coefficient of C0 composite decreases to 22.0% while that of C10 composite still remains 43.0%. Better thermal stability was also found for the EPDM composite containing CNTs as compared to that without CNTs. In a word, CNTs is highly effective antioxidant filler for the EPDM, and thus would facilitate the wide application of EPDM resistance to thermal-oxidative aging.

## Figures and Tables

**Table 1:** Recipe of EPDM/silica/CNTs composites (unit: per hundred rubber, phr).

Sample	C0	C2	C4	C8	C10
EPDM	100	100	100	100	100
Silica	40	38	36	32	30
CNT	0	2	4	8	10
ZnO	8	8	8	8	8
MgO	4	4	4	4	4
4010NA ^a^	2	2	2	2	2
TAIC ^b^	2	2	2	2	2
DCP ^c^	4.5	4.5	4.5	4.5	4.5

^a^ N-[4-(phenylamino)phenyl]maleimide; ^b^ triallyl isocyanurate; ^c^ dicumyl peroxide.

**Table 2:** Mechanical properties of EPDM/silica/CNTs composites.

Properties	C0	C2	C4	C8	C10
Hardness (Shore A)	66	71	72	74	75
Stress at 100% strain (MPa)	1.7	2.5	3.4	4.8	6.5
Elongation at break (%)	863	748	568	382	328
Tensile strength (MPa)	19.8	19.9	18.3	16.0	16.5

**Table 3:** TGA parameters for unaged and aged EPDM composites (aging condition: 150 °C × 168 h).

Sample	*T*_5%_ (°C)	*T_max_* (°C)	Residue Mass (%)
unaged C0	430.8 ± 1.2	488.5 ± 0.2	33.6 ± 0.2
aged C0	442.7 ± 1.1	486.0 ± 0.3	34.3 ± 0.3
unaged C10	434.6 ± 0.9	488.3 ± 0.3	34.1 ± 0.2
aged C10	447.6 ± 1.0	489.6 ± 0.4	34.5 ± 0.1

## Data Availability

Not applicable.
